# Supplementary material for: The Systems Biology Research Tool: evolvable open-source software
Source: BMC Syst Biol. 2008 Jun 29;2:55. doi: 10.1186/1752-0509-2-55 (PMC2446383; doi:10.1186/1752-0509-2-55)
Supplement: Additional file 1 — SBRT Archive. An archive of the current version of the Systems Biology Research Tool. [file 1752-0509-2-55-S1.zip › sbrt-1.4.0/doc/developers_guide/api/sbrt/kernel/math/QuadraticExpr.html]

QuadraticExpr


|  |  |  |
| --- | --- | --- |
| **Overview** \| **Package** \| **Class** \| **Use** \| **Tree** \| **Deprecated** \| **Index** \| **Help** | |  |
| **PREV CLASS**   **NEXT CLASS** | **FRAMES**    **NO FRAMES**     **All Classes** |  |
| SUMMARY: NESTED \| FIELD \| CONSTR \| METHOD | DETAIL: FIELD \| CONSTR \| METHOD |  |


---


## sbrt.kernel.math Interface QuadraticExpr<V>

**Type Parameters:**: `V` - the variable type.

**All Superinterfaces:**: MathExpr<V>, Polynomial<V>

---

``` public interface QuadraticExpr<V> extends Polynomial<V> ```

This interface is used to represent quadratic expressions. It has not been
fully implemented.

**Author:**
:   This interface was written and documented by Jeremiah Wright
    while in the Wagner lab.

---

| **Nested Class Summary** | |
| --- | --- |
| `static interface` | `QuadraticExpr.Term<V>`             This interface is used to represent the terms of quadratic expressions. |


**Method Summary**

**Methods inherited from interface sbrt.kernel.math.Polynomial**

`equals, getConstant, getTermArray, hashCode, plugInNumbers, terms, toString`

**Methods inherited from interface sbrt.kernel.math.MathExpr**

`getVariables, hasAllVariables, hasVariable, hasVariables, variables`

---


|  |  |  |
| --- | --- | --- |
| **Overview** \| **Package** \| **Class** \| **Use** \| **Tree** \| **Deprecated** \| **Index** \| **Help** | |  |
| **PREV CLASS**   **NEXT CLASS** | **FRAMES**    **NO FRAMES**     **All Classes** |  |
| SUMMARY: NESTED \| FIELD \| CONSTR \| METHOD | DETAIL: FIELD \| CONSTR \| METHOD |  |


---
